# Mobile stroke units services in Germany: A cost‐effectiveness modeling perspective on catchment zones, operating modes, and staffing

**DOI:** 10.1111/ene.16514

**Published:** 2024-11-06

**Authors:** Johann S. Rink, Kristina Szabo, Carolin Hoyer, Jeffrey L. Saver, May Nour, Heinrich J. Audebert, Wolfgang G. Kunz, Matthias F. Froelich, Armin Heinzl, Andrej Tschalzev, Jens Hoffmann, Stefan O. Schoenberg, Fabian Tollens

**Affiliations:** ^1^ Department of Radiology and Nuclear Medicine University Medical Centre Mannheim, University of Heidelberg Mannheim Germany; ^2^ Department of Neurology University Medical Centre Mannheim, University of Heidelberg Mannheim Germany; ^3^ Department of Neurology UCLA Stroke Center, University of California Los Angeles California USA; ^4^ Departments of Neurology and Radiology Ronald Reagan UCLA Medical Center Los Angeles California USA; ^5^ Department of Neurology and Center for Stroke Research Berlin Charité–Universitätsmedizin Berlin Berlin Germany; ^6^ Department of Radiology University Hospital, LMU Munich Munich Germany; ^7^ University of Mannheim Mannheim Germany; ^8^ Institute for Enterprise Systems, University of Mannheim Mannheim Germany

**Keywords:** acute stroke care, acute stroke therapy, computed tomography, economic evaluation, mobile stroke unit, telemedicine

## Abstract

**Background and Purpose:**

Investigating the cost‐effectiveness of future mobile stroke unit (MSU) services with respect to local idiosyncrasies is essential for enabling large‐scale implementation of MSU services. The aim of this study was to assess the cost‐effectiveness for varying urban German settings and modes of operation.

**Methods:**

Costs of different operating times together with different personnel configurations were simulated. Different possible catchment zones, ischemic stroke incidence, circadian distribution, rates of alternative diagnoses, as well as missed cases were incorporated to model case coverage and patient numbers. Based on internationally reported clinical outcomes of MSUs, a 5‐year Markov model was applied to analyze the cost‐effectiveness for the different program setups.

**Results:**

Compared with standard stroke care, MSUs achieved an additional 0.06 quality‐adjusted life years (QALYs) over a 5‐year time horizon. Assuming a catchment zone of 750,000 inhabitants and 8 h/7 day operation resulted in an incremental cost‐effectiveness ratio (ICER) of €37,182 per QALY from a societal perspective and €45,104 per QALY from a healthcare perspective. Lower ICERs were possible when coverage was expanded to 16 h service on 7 days per week and larger populations. Sensitivity analyses revealed that missing ischemic strokes significantly deteriorated economic performance of MSU.

**Conclusions:**

Major determinants of cost‐effectiveness should be addressed when setting up novel MSU programs. Catchment zones of more than 500,000–700,000 inhabitants and operating times of at least 12–16 h per day, 7 days per week could enable the most cost‐effective MSU services in the German healthcare system.

## Graphical abstract

The analysis explored the long‐term economic effects of mobile stroke unit (MSU)‐based care in the German healthcare system. When setting up novel MSU programs, catchment zones of >500,000–700,000 inhabitants and operating times of at least 12–16 h per day on 7 days per week are preferable and could enable the most cost‐effective MSU services.

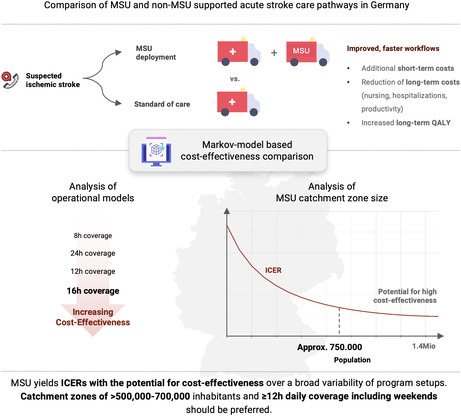

## INTRODUCTION

Stroke is a leading cause of disability and mortality and a great socioeconomic burden [1]. In recent years there have been considerable advances in acute stroke management aimed at maximizing the chance of timely administration of intravenous thrombolysis (IVT) and endovascular therapy (EVT). Even though there is evidence that early therapy initiation reduces post‐stroke disability [2], less than 5% of patients receive IVT within the first 60 min after stroke onset in many settings [3], and various pre‐ and in‐hospital delays compromise workflow speed in stroke care [4].

Mobile stroke units (MSUs) are specialized ambulances capable of performing head computed tomography (CT) allowing for improved triage and pre‐hospital therapy initiation [5]. This concept has gained considerable interest as multiple prospective controlled studies in Europe and the United States (US) have demonstrated a significant reduction of post‐stroke disability and mortality [6, 7]. Yet, there is no standard approach to conceptualization of MSU services, leading to highly individualized approaches regarding hardware, staffing, catchment zones, and alarming strategy, and to heterogeneous dispatch, utilization, and IVT rates [8].

While the European Stroke Organisation (ESO) recommends the implementation of MSUs [9], widespread adoption is restricted by barriers such as considerable investment expenditures [10, 11, 12] and the necessity for highly qualified personnel. A major objective when implementing the guidelines is to strike a balance between clinical benefits from improved stroke care and the demand for scarce healthcare system resources.

We aimed to design an economic model and assess the cost‐effectiveness of MSU services for varying modes of operation under urban conditions in Germany.

## METHODS

Cost‐effectiveness modeling was based on input parameters taken from the literature and data from local hospital and emergency medical services (EMS) accounting departments. All used parameters and probability distributions are reported in eTables S1–S9. Recommendations on reporting of economic evaluations were considered according to the CHEERS checklist (eTable S13) and input parameters were validated where possible (eTable S10, eFigure S5). No human data were analyzed, hence institutional review board (IRB) approval was waived.

### Economic model

**FIGURE 1:**
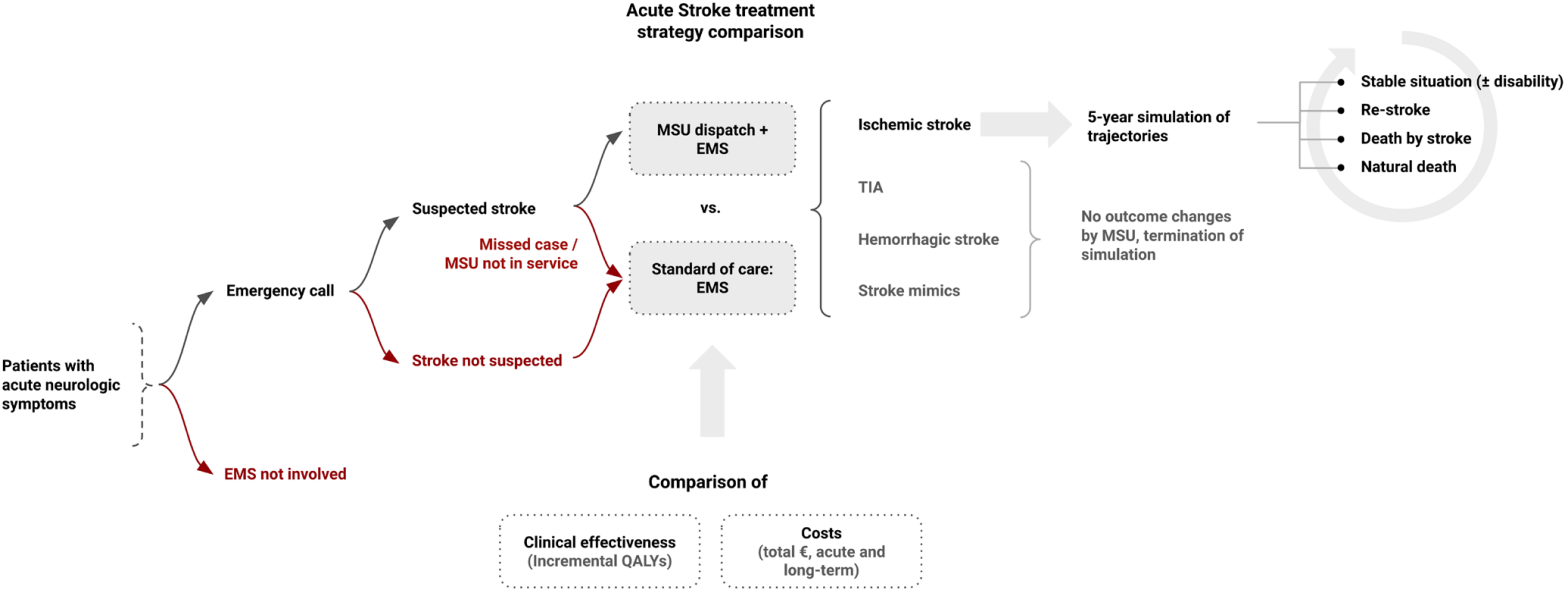
Comparison of acute stroke care pathways. Patient pathways upon entering the model. A mobile stroke unit (MSU) is deployed when a stroke is suspected at dispatch and it is available. MSU‐based care is compared with conventional care by emergency medical services. The number of patients is defined by the population in the catchment zone and incidence rates. Improvement of outcomes is only assumed for ischemic stroke patients treated by MSU. Therefore, a time horizon of 5 years was simulated for these patients, including quality‐adjusted life years and costs of long‐term care. EMS, emergency medical services; QALY, quality‐adjusted life year; TIA, transient ischemic attack.

Modeling software TreeAge Pro 2020 (TreeAge, Williamston, MA, USA) was used to design a Markov model comparing the strategies of conventional acute stroke care versus MSU‐based stroke management. Follow‐up was simulated over a timeframe of 5 years with a cycle length of 1 year, representing a timeframe which can be foreseen in terms of costs and outcomes (Figure 1). The disease states ischemic stroke, transitory ischemic attack (TIA), hemorrhagic stroke, and stroke mimic were included.

Based on different German MSU trials, the average patient age was set at 72.5 years [6, 13, 14, 15] and only patients without prior strokes were assumed to enter the model. The number of patients presenting with ischemic stroke, hemorrhagic stroke, TIA, and stroke mimics was estimated based on German incidence rates [16], reports on the rate of stroke mimics on MSUs [17], and simulated catchment zone sizes, which was assumed to be 750,000 inhabitants in the base case, representing a realistic average population of MSU sites in Germany. The number of stroke cases was reduced by fractions of patients who did not contact rescue services [18] or would not fulfill MSU alarming criteria. Chances of stroke misidentification at dispatch level was estimated at 37%, based on various reports indicating very heterogeneous dispatcher sensitivity between 48.9% and 77.7% [19, 20, 21, 22]. Another 8% of cases were estimated to be missed due to logistic problems [8], which increased by 2.5% per every 100,000 inhabitants covered due to simultaneous stroke events. Details are provided in the supplementary material.

An increased rate of IVT treatments (12.1%) was assumed for MSU, and the need for secondary interhospital transfers was obviated by MSU [23, 24]. Probabilities of ending up on a specific level of disability after a stroke event were obtained from both the German B_PROUD and the US BEST‐MSU studies [6, 7]. Since outcome data for hemorrhagic stroke patients or stroke mimics are not available for MSU services to date, outcome improvement by MSU was simulated only for ischemic stroke patients, and not for hemorrhagic stroke patients nor stroke mimics. Therefore, the long‐term simulation only followed up ischemic stroke patients.

Average per‐patient costs and outcomes of MSU service and standard stroke care were calculated. The difference in costs (incremental costs) and outcomes (incremental effectiveness) was synthesized in the incremental cost‐effectiveness ratio (ICER). Cost and outcomes were discounted by 3% annually [25].

### Transition probabilities

For the 5‐year simulation, ischemic stroke patients could either stay in the health state of stable post‐stroke disability according to the reported distribution of functional impairment for both strategies, suffer a further stroke leading to the same or deteriorated disability levels, or enter the health state “death” (eFigure S1). Specific mortality rates as well as time‐dependent rates of stroke recurrence were considered [13, 15].

### Costs

The decision model‐supported cost‐effectiveness analysis followed both the German healthcare and societal perspectives, and all cost parameters are provided in the supplementary material. The German consumer price index (CPI) was used to inflate costs to 2021 € [14]. Capital costs and running costs were estimated based on data from the Berlin MSU [26] and cost data from the hospital and local EMS. Hardware costs in € were calculated for a depreciation timeframe of 6 years. Total staffing costs for different modes of operation were estimated based on local hospital and EMS data, including a general hospital administration overhead of 22%, similar to the established overhead for research funding and additional training costs. The team consists of one paramedic, a physician (neurologist), a radiology technician, a remote radiologist (not exclusive to MSU), and a physician project manager (50%). The model included both costs for EMS transportation and MSU management in the MSU group, as a rendezvous system with a regular ambulance for transportation was assumed. Direct hospital costs of acute ischemic stroke treatment were added according to modified Rankin Scale (mRS) level.

Long‐term post‐stroke costs according to functional status were estimated by calculating the costs of stroke‐induced additional days of hospitalization per year from the healthcare system perspective. To model the societal perspective, the insurance‐covered fraction of costs for long‐term home care or nursing was included as well as family‐covered nursing costs and productivity losses due to morbidity and mortality according to the human capital approach.

### Utility levels

Outcomes were reported in 90‐day post‐stroke mRS states by the investigators of the source studies. They were converted into quality‐adjusted life years (QALYs) via the German utility values [27].

### Cost‐effectiveness thresholds

There are no official willingness‐to‐pay (WTP) thresholds in Germany [28]. The World Health Organization defined the approach of using the gross domestic product (GDP) per capita, which yields a threshold of €47,901 per QALY gained (1× GDP) for Germany [29].

### Sensitivity analysis

Deterministic two‐way sensitivity analysis was used to assess the model stability when the number of missed stroke cases were varied and different operational models were applied (eFigure S2).

Probabilistic sensitivity analysis was utilized to determine the model stability with 10,000 Monte Carlo iterations when multiple input parameters were varied according to their assigned probability distributions.

Scenario sensitivity analyses for different operation modes ranging from 5 to 7 days per week, 8, 12, 16 or 24 h per day were included, based on the circadian distribution of strokes [30], for which a maximum of 300 operational days per year was assumed, based on experience from the Berlin MSU program. Moreover, an analysis accounting for different possible catchment zone areas was included.

## RESULTS

### Patient‐level MSU costs

For the base case, short‐term patient‐level costs for MSU deployment amounted to €10,040 in ischemic stroke patients and to €3620 for all patients managed by MSU. Some 15.8% of total costs were investment expenditures, 37.6% were running costs, whereas 46.6% were staffing costs. Average MSU costs per ischemic stroke patient were 11.1% lower when teleneurological assessment was applied.

### Cost‐effectiveness results

For a population of 750,000 inhabitants and a coverage of 8 h on 7 days in a year, a total of 124 ischemic stroke patients are managed by the MSU. Average discounted costs and QALYs are reported in Table 1. For all the patients that the MSU was dispatched to, incremental costs over a time frame of 5 years amounted to €2548/€2101 and 0.06/0.06 incremental QALYs were accrued, and the resulting ICERs were €45,104/€37,182 per QALY gained (healthcare perspective/societal perspective).

**TABLE 1:** Cumulative average costs and quality‐adjusted life years for healthcare and societal perspectives.

Strategy	Average discounted costs, healthcare perspective (€)	Average discounted QALYs, healthcare perspective	Average discounted costs, societal perspective (€)	Average discounted QALYs, healthcare perspective
Standard care	17,923	2.23	33,341	2.23
MSU‐based stroke care	20,471	2.29	35,442	2.29

*Note*: Costs and QALYs are reported for a population of 750,000 in the base case analysis.

Abbreviations: MSU, mobile stroke unit; QALY, quality‐adjusted life year.

### Scenario sensitivity analyses on operational models and catchment zones

**FIGURE 2:**
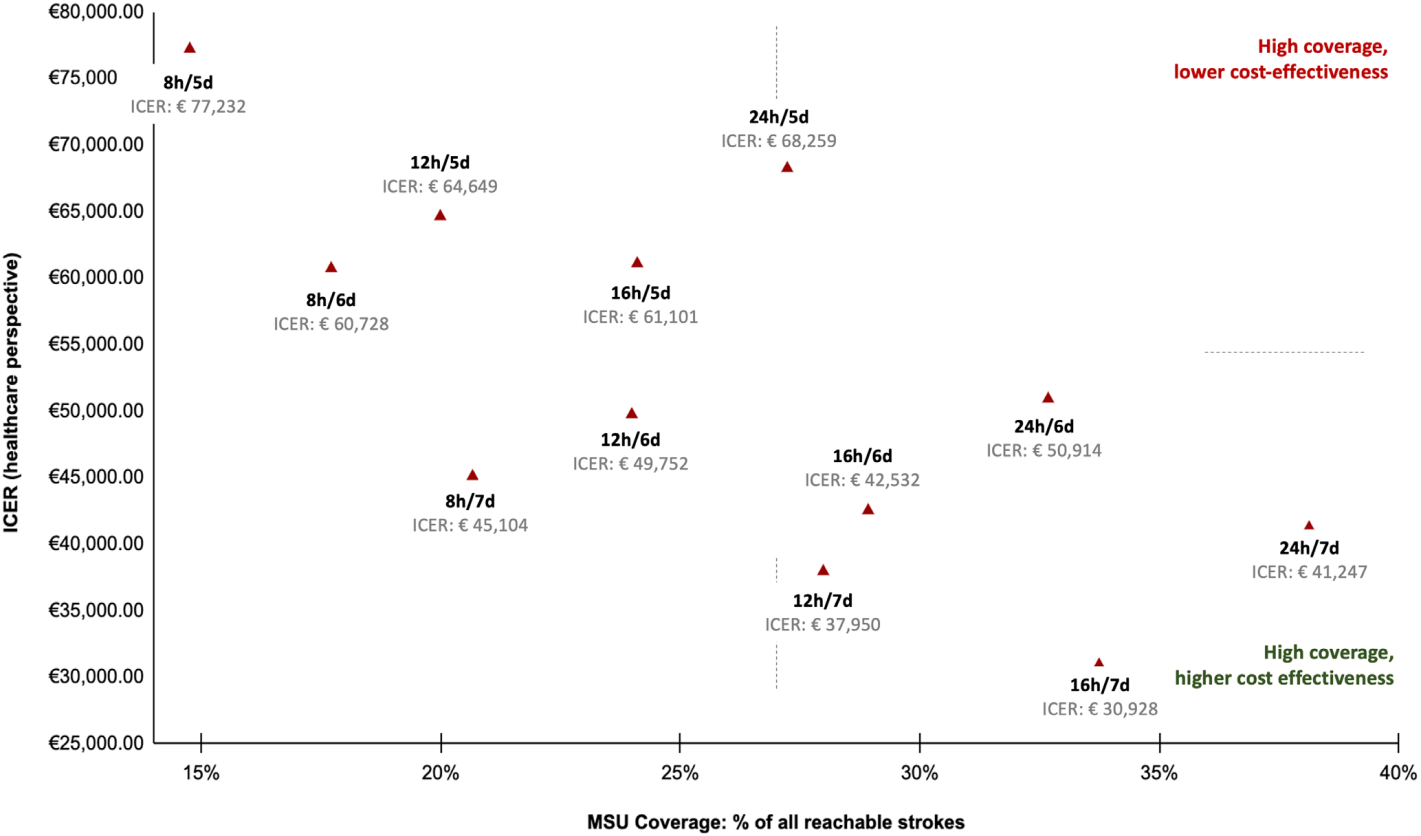
Cost‐effectiveness simulation for different operating modes. For 750,000 inhabitants, incremental cost‐effectiveness ratios (ICERs) of 12 different operating modes were computed from the healthcare perspective in relation to mobile stroke unit (MSU) stroke coverage. There are substantial differences in economic performance of the models, suggesting that the 16 h/7 day and 12 h/7 day models offer higher coverage at lower ICERs, and that 8 h coverage models lead to comparably poor coverage at relatively higher costs.

For a population of 750,000 inhabitants, ischemic stroke case coverage by MSU ranged from 16.8% (8 h/5 days) to 42.5% (24 h/7 days) (Figure 2, eTable S11). The model of 16 h/7 days yielded the lowest ICER (€30,928 per QALY, healthcare perspective) and the model of 8 h/5 days led to the highest ICER (€77,232 per QALY, healthcare perspective).

**FIGURE 3:**
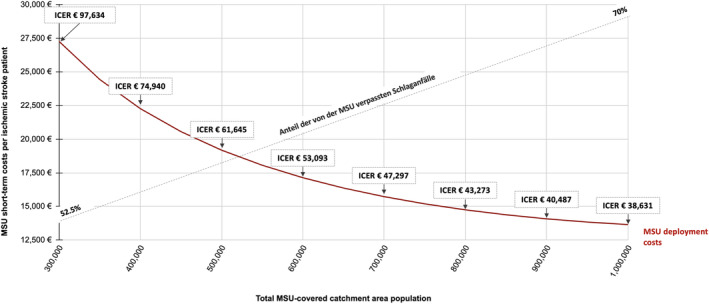
Mobile stroke unit (MSU) short‐term costs and incremental cost‐effectiveness ratios (ICERs) according to catchment zone size. Short‐term costs of MSU deployment per ischemic stroke patient and corresponding ICERs from the healthcare perspective of 5‐year modeling are displayed for varying catchment zone populations for an 8 h/7 day model. Higher catchment zone population leads to lower costs per patient and lower ICERs. Optimistic and pessimistic scenarios display a range of possible cost outcomes.

ICER significantly decreased when more inhabitants were included (Figure 3 and Table 2). The catchment zone population needed to exceed about 500,000–700,000 inhabitants for the MSU service to yield ICER values below the WTP‐threshold in healthcare and societal perspectives.

**TABLE 2:** Costs and cost‐effectiveness according to catchment zone population.

Catchment zone population	Missed IS cases (%)	IS patients managed by MSU per year	MSU short‐term deployment cost per IS patient (€)	ICER (€/QALY), healthcare perspective (€)	ICER (€/QALY), societal perspective (€)
200,000	51.25	43.7	28,424	143,716	135,794
300,000	52.50	62.7	24,791	97,634	89,712
400,000	55.0	79.7	15,602	74,940	67,018
500,000	57.5	94.7	13,124	61,645	53,723
600,000	60.0	107.8	11,529	53,093	45,171
700,000	62.5	119.0	10,449	47,297	39,375
800,000	65.0	128.2	9669	43,273	35,351
900,000	67.5	135.4	9179	40,487	32,565
1,000,000	70.0	140.7	8833	38,631	30,709

*Note*: Costs of MSU deployment, ICER, and rate of missed strokes for varying catchment zone populations.

Abbreviations: ICER, incremental cost‐effectiveness ratio; IS, ischemic stroke; MSU, mobile stroke unit; QALY, quality‐adjusted life year.

### Sensitivity analysis for the base case

Two‐way deterministic sensitivity analysis demonstrated decreased net monetary benefits when increasing the number of missed strokes. Choice of operation model also has significant relevance for economic benefits (eFigure S2).

**FIGURE 4:**
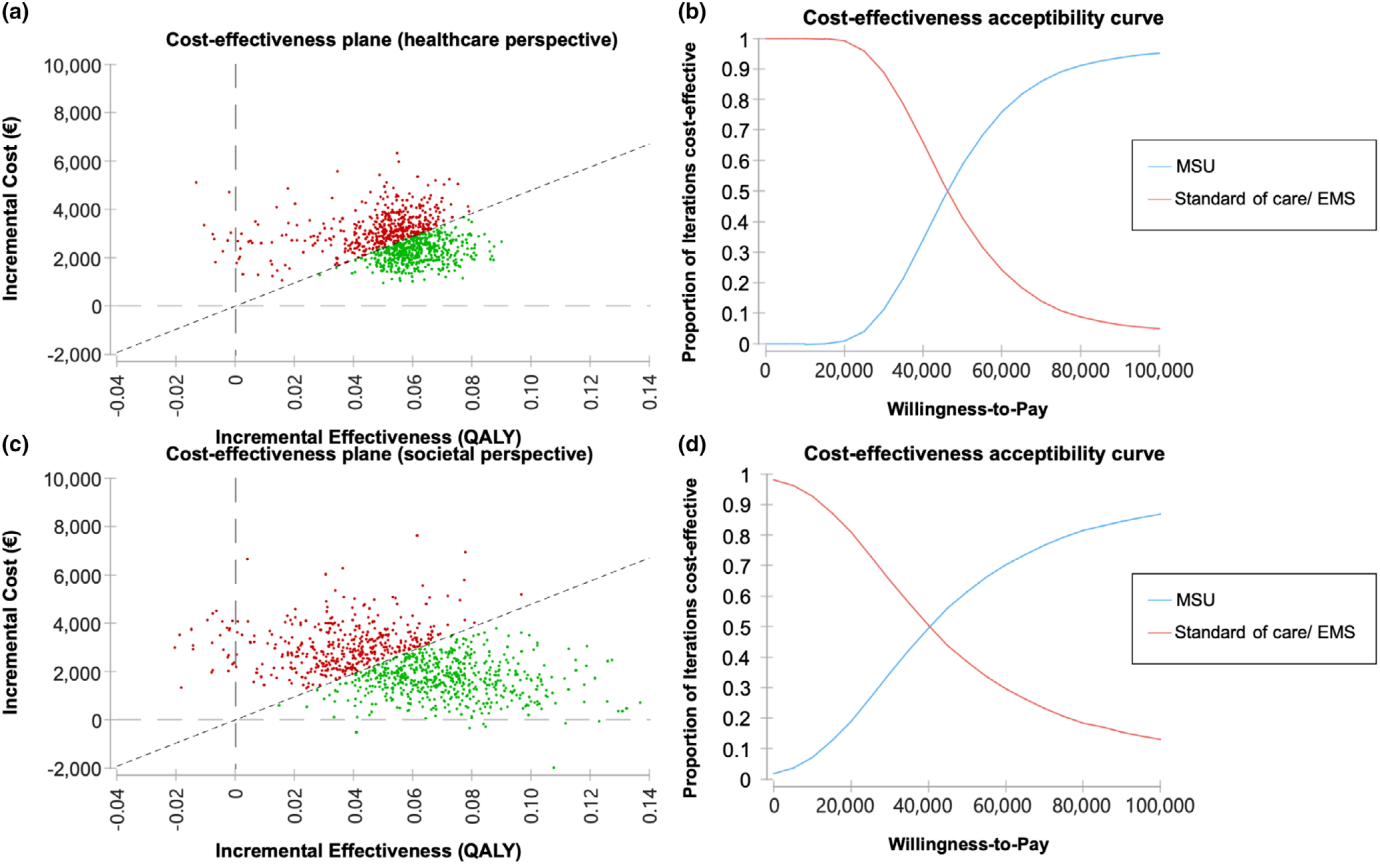
Probabilistic sensitivity analyses.The figure panel shows the results of the probabilistic sensitivity analysis of the base case based on 10,000 Monte Carlo iterations based on probability distributions of the input parameters. The cost‐effectiveness plane demonstrates the distribution of the iterations below (green) and above (red) an incremental cost‐effectiveness ratio (ICER) of €47,901 per ICER, for a healthcare system perspective (a) and societal perspective (c). Corresponding acceptability curves for varying willingness‐to‐pay thresholds (b and d). EMS, emergency medical services; MSU, mobile stroke unit; QALY, quality‐adjusted life year.

Probabilistic sensitivity analysis confirmed the stability of the economic model for a variety of input parameters. At a WTP of €47,901 per QALY gained, 55.4%/58.9% of iterations (healthcare perspective/societal perspective) were found to be cost‐effective (Figure 4).

## DISCUSSION

Our economic model demonstrates the potential of creating cost‐effective MSU services in Germany. Economic analyses, including the most recent clinical evidence, and modeling plausible real‐world scenarios in Germany enable a differentiated understanding of key determinants for cost‐effective MSU operation.

Various operating modes have been described, ranging from 8 h/5 days to 24 h/7 days [31, 32]. Within this analysis, models with ≥12 h daytime coverage including weekend service exhibit superior economic performance and should therefore be preferred to enable the most efficient use of healthcare resources. Notably, we found MSU coverage of ischemic stroke patients to vary from <20% to 40% depending on operating hours.

There seems to be a substantial heterogeneity concerning investment expenditures of MSU within the various projects ranging from €432,820 [33] to €1,139,000 [26]. As staffing costs dominate the overall costs per patient, their reduction seems promising. Teleneurology has been shown to be clinically feasible [34] and led to improved overall costs within this analysis. Even though its implementation can be challenging due to regulatory frameworks and the need to minimize possible adverse effects causing further workflow delays, it offers the perspective to operate more units by centralizing neurological assessments more efficiently.

A large number of different catchment zone population sizes has been reported ranging from 184,000 in the UK to up to >2,000,000 in Houston, Texas, USA [35, 36]. Projects operating under rural conditions such as in Canada [37] and Norway [38] typically cover large geographical regions whereas others serve densely populated, small urban areas (e.g., New York City, New York, USA) [39]. With only limited early evidence for urban conditions [40], optimal MSU catchment zone size remains unknown. This analysis provides the first insights into the relation between potential catchment zone sizes in Germany and economic outcomes. The lack of real‐world outcome data for very small and very large populations should be considered when interpreting the results.

Sensitivity analysis revealed that the rate of missed strokes plays a critical role when estimating the number of stroke patients and the resulting cost‐effectiveness for an MSU service. Missing strokes may be due to direct referral to emergency departments, non‐identification, simultaneous cases, and logistical problems at the MSU level. Our analysis was the first to directly include this factor in cost‐effectiveness modeling. To increase the low coverage of ischemic stroke patients, efforts in dispatcher training may have significant relevance, which is further underlined by data from PHANTOM‐S [41] and B_PROUD [6] studies which indicated repetitive training to be effective.

The recent study of Gonçalves et al. reported an ICER of €40,984 per QALY for an MSU service in Berlin, Germany (societal perspective) [26]. This analysis yields ICERs in the same range, but relies on a different methodology, a different distinct set of input parameters and additional inclusion of post‐stroke hospitalization costs, family‐covered nursing costs, and productivity losses. It confirms the validity of the ICERs reported previously, underlining the downstream economic value of MSU‐based stroke care from a societal perspective. It is important to note that in the absence of well‐established WTP thresholds in Germany, the main strategic direction lies in optimization of the services with respect to maximizing the covered population and its economic performance.

Widespread adoption of the MSU approach necessitates reimbursement [42]. As the treatment spectrum of MSU might be extended in the future [17], reimbursing MSU services in all patients who receive complete diagnostic workup in the MSU seems most reasonable, which was €3620 per patient in this analysis. Considerable variation in patient‐level costs should be expected depending on local setup.

The presented analysis has to be interpreted with respect to its limitations. Input parameters were derived from the international literature, partly from different healthcare systems; however, with careful consideration to achieve realistic estimates. Nevertheless, the uncertainty regarding the proportion of stroke patients missed by MSU could not be precluded due to the lack of sufficient data. Rates of missed simultaneous cases were extrapolated based on stroke incidence and population size. The potential of MSUs for earlier treatment of ICH and large vessel occlusion via EVT was not taken into consideration. In modeling of 5‐year post‐stroke costs, even though a conservative approach was applied, imprecision might be present. The sum of these risks was addressed by modeling deterministic and probabilistic sensitivity analyses. Essentially, the limited experience with economic evaluations in the German healthcare system restricts the ability to compare MSUs with other acute stroke care innovations. As the clinical outcomes were derived from urban projects in Germany and the US, the transferability of the findings to projects operating under rural conditions is limited.

In conclusion, this analysis suggests that healthcare resources can be used most efficiently when urban MSU services in Germany cover catchment zones of more than 500,000–700,000 inhabitants and operate for ≥12 h daily including weekends, which offers the best opportunities regarding cost‐effectiveness. Detailed data on missed strokes and long‐term costs need to be acquired from running MSU projects to improve economic understanding. Optimization of dispatch and alarming workflows and the utilization of telemedicine will represent cornerstones of successful MSU programs.

## AUTHOR CONTRIBUTIONS

**Johann S. Rink:** Conceptualization; writing – original draft; validation; data curation; project administration; writing – review and editing; visualization; investigation; resources; supervision. **Kristina Szabo:** Conceptualization; writing – review and editing; resources; supervision; validation. **Carolin Hoyer:** Conceptualization; writing – review and editing; supervision; validation. **Jeffrey L. Saver:** Writing – review and editing; validation; supervision. **May Nour:** Validation; writing – review and editing; supervision. **Heinrich J. Audebert:** Writing – review and editing; supervision; validation. **Wolfgang G. Kunz:** Methodology; writing – review and editing; validation; conceptualization. **Matthias F. Froelich:** Validation; writing – review and editing; supervision. **Armin Heinzl:** Writing – review and editing; validation; supervision; resources; funding acquisition. **Andrej Tschalzev:** Writing – review and editing; validation; methodology. **Jens Hoffmann:** Writing – review and editing; validation. **Stefan O. Schoenberg:** Supervision; resources; writing – review and editing; funding acquisition. **Fabian Tollens:** Writing – original draft; methodology; software; formal analysis; data curation; visualization; conceptualization; investigation; validation; writing – review and editing.

## CONFLICT OF INTEREST STATEMENT

SOS: The Department of Radiology and Nuclear Medicine has general research agreements with Siemens Healthineers. JLS has received, for service on clinical trial steering committees and data and safety monitoring boards advising on rigorous study design and conduct, hourly payments from Medtronic, Abbott, NeuroVasc, Phillips Medical, Bayer, Biogen, Roche, BrainsGate, BrainQ, CSL Behring, and Occlutech, and stock options from Rapid Medical and QuantalX. Other authors: no conflicts of interest.

## Supporting information


**Data S1.** Supporting information.

## Data Availability

The data that support the findings of this study are available from the corresponding author upon reasonable request.
