# Supplementary material for: Mobile stroke units services in Germany: A cost‐effectiveness modeling perspective on catchment zones, operating modes, and staffing
Source: Eur J Neurol. 2024 Nov 6;32(1):e16514. doi: 10.1111/ene.16514 (PMC11622509; doi:10.1111/ene.16514)
Supplement: Supplementary file 1 — Data S1. Supporting information. [file ENE-32-e16514-s001.docx]

#

#

Supplementary Materials

[Model details 2](#_xrh5bhb5pdqg)

[Details on the 5-year Markov model 2](#_2wxaa1dnttoj)

[MSU personnel costs estimation 4](#_73x10opjqbqg)

[Estimation rate of MSU-missed strokes 7](#_vbh0h31cb4ez)

[Estimation of stroke-induced long-term inpatient healthcare costs 8](#_nnk83wlqox7p)

[Estimation of long-term stroke-induced nursing costs 8](#_i478saxcwaxp)

[Estimation of long-term stroke-induced productivity losses 10](#_vavu2ledmcf2)

[Long-term informal care needs 10](#_axln4wn3muuz)

[Economic modeling input parameters 10](#_1cdabjer2sw9)

[Results: Operation modes, coverage, catchment zones and corresponding ICERs 15](#_jmbnjekhcdb6)

[Adherence to established reporting standards 18](#_f8xbf0k275q7)

[CHEERS Checklist 18](#_sss97cn842le)

[Size of catchment zones reported by international MSU projects and association of driving distance and catchment zone population in Mannheim 20](#_jflqyv1hva3i)

[Comparison of outcomes with other MSU cost-effectiveness analyses 22](#_fkv2ij9wmcz9)

[Validation of modeled survival rates 23](#_36nv1ls483de)

[Supplemental References 24](#_ic41yqdh5bqc)

##

## Model details

### Details on the 5-year Markov model


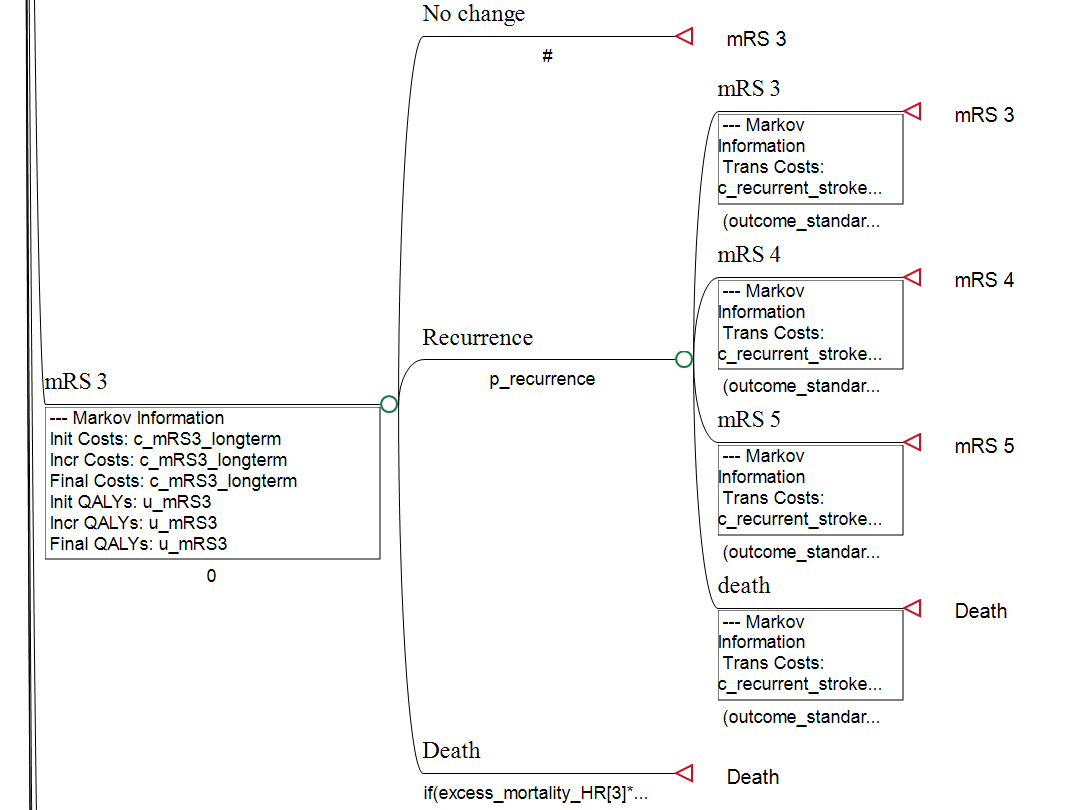


**eFigure 1: Markov Model capturing the course of stroke patients.**Representative part of the Markov Model that displays the possibility of recurrence of stroke or death of patients in mRS 3 state. Following recurrence of stroke, the same level of disability or deterioration is possible.

The 5-year Markov model succeeded the acute care simulation. Green nodes represent chance nodes, whereas red triangles represent terminal nodes. After suffering an initial stroke, different patient pathways were simulated in a decision-tree that included the options of MSU-based care, IVT and short-term recurrence of stroke within the first year. Depending on the post-stroke mRS level, patients entered different Markov states. Each mRS state was represented by a specific set of costs and associated quality of life that was converted into QALYs based on the cycle length of one year. Patients could either remain on their mRS level, die over time or suffer an additional stroke which was assumed based on the time-dependent chance of re-stroke after the initial event (no other factors influencing re-stroke chance). Suffering an additional stroke meant to stay on the level of disability or to change to a higher mRS category.

| **Parameter** | **Value** | **Distribution in probabilistic sensitivity analysis** | **Source** |
| --- | --- | --- | --- |
| **Mobile Stroke Unit (MSU) metrics - assumptions** | | | |
| Incidence of ischemic strokes/ hemorrhagic strokes/ TIA in Germany per 100,000 inhabitants per year | 194.42 /  37.36 /  124.10 | dirichlet | Estimate based on reported incidence from Erlangen, Germany ^1^ |
| Proportion of stroke patients treated by EMS | 62% | beta | Fladt et al 2019 ^2^ |
| Proportion of alternative diagnoses (stroke mimics) | 50% | beta | Estimate, based up Fassbender et al 2023 ^3^ |
| Proportion of stroke mimics receiving full MSU management including CT scan | 68% | beta | Calculated upon Fassbender et al 2023 ^3^ |
| Percentage of stroke patients within time windows of 8h/12h/16h covering most cases according to circadian stroke incidence | 8h: 10 a.m. - 6 p.m.: 55.2%  12h: 10 a.m. - 10 p.m.: 76.8%  16h: 8 a.m. - 12 p.m.: 93.1% | beta | Wroe et al 1991 ^4^ |
| Proportion of missed stroke cases | 37% misidentification at dispatch  8.0% MSU logistic problems and simultaneous events)  2.5% per 100,000 inhabitants covered (simultaneous cases) | beta | Estimate based on Ellens et al 2022 ^5^, Matilla et al 2019 ^6^ Mould-Millman et al 2018 ^7^ Nour et al 2020 ^8^ Eliakundu et al 2022 ^9^ and Bohm et al 2018 ^10^ |
| Proportion of patients with ischemic strokes and TIAs being tPA eligible and receiving tPA (MSU / EMS) | 60.2% /  48.1 % | beta | Ebinger et al 2021 ^11^ |
| Proportion of patients with stroke mimics being tPA eligible and receiving tPA (MSU / EMS) | 17.3% /  17.0% | beta | Estimate based on Grotta et al 2021 ^12^ |
| MSU running days per year (including weekends, excluding holidays, maintenance and training time) | 300 | beta | Estimate based on local regulations and on experience from the Berlin Mobile Stroke Unit |
| Proportion of ICH patients who require interhospital transfer (MSU / EMS) | 0 / 40% | beta | Estimate based upon Helwig et al 2021 ^13^ and Backhaus et al 2015 ^14^ |

**eTable 1: MSU metrics**

This table displays the model input parameters concerning metrics of MSU operation.

### MSU personnel costs estimation

In the base case analysis, MSU was assumed to be staffed by a team of a Neurologist, Radiology technician and one EMS paramedic and a remote radiologist, covering the unit via telemedicine. This set-up was chosen as the most probable one based on German regulations at the time the analysis was conducted.

Official wage agreements are in place countrywide and regulate the costs of physicians (“Tarifverträge der Länder - Ärzte Unikliniken”) and radiology technicians (Tarifververtrag Öffentlicher Dienst E7 level 3). Paramedics can be expected to be compensated as Notfallsanitäter, salaries are noted in the Tarifvertrag Öffentlicher Dienst E9c. As salaries vary depending on marital status, experience and time of employment, averages were calculated by the local hospital controlling department to obtain realistic costs.

Mandatory extra benefits for changing shifts (€ 40 / month), night shifts (20% per hour 9PM-6AM), Saturday shifts (35% 1PM-9PM), Sunday shifts (25% per hour), holidays (25% per hour) were considered according to official tariffs.

To estimate costs for teleradiology and teleneurology, extrapolations of average case numbers were conducted based on literature. A fraction of 9% staffing costs for both a radiologist and a neurologist were added to personnel costs depending on the scenario which was calculated. The rest of personnel costs were estimated to be covered by regular compensation within the hospital departments.

As an established German research funding overhead, 22% additional personnel costs were added as administration costs^15^, which from our experience represents a well-established average for research funding in Germany. Project management was assumed to be performed by a physician functioning as a medical director (part-time, 50-75%, depending on staffing model).

Additional training was assumed necessary for neurologist and radiology technician, and the costs of courses for training was added.

For simulation of teleneurology, a fraction of 9% of neurology physician costs were included based on detailed simulation of average time spent on MSU consultations per workday.

| **Detailed personnel costs 8h7 days operational model** |  |
| --- | --- |
| Wage costs MSU teams (running period) | €412,600 |
| Project management wages (75% attending) | €101,979 |
| Wage costs MSU teams (training period) | €38,43 |
| Training course costs | €5,35 |
| Costs for extra benefits | €20,281 |
| **Total** | **€578,649** |

**eTable 2: Exemplary cost calculation for the personnel assuming a 8h7day model (in thousands €)**

| **Operation model** | **Annual personnel costs** |
| --- | --- |
| **8h5d** | 347,755 € |
| **8h6d** | 514,877 € |
| **8h7d (base case)** | 578,649 € |
| **8h7d Teleneurology** | 440,050 € |
| **12h5d** | 505,519 € |
| **12h6d** | 678,826 € |
| **12h7d** | 795,376 € |
| **16h5d** | 678,475 € |
| **16h6d** | 756,421 € |
| **16h7d** | 831,055 € |
| **24h5d** | 1,008,207 € |
| **24h6d** | 1,202,122 € |
| **24h7d** | 1,464,904 € |

**eTable 3: Personnel costs for different operational models**

Costs were calculated based on real-world data from the local hospital accounting department and local EMS accounting department, based on official tariffs.

### Estimation rate of MSU-missed strokes

The MSU was assumed to operate within a specific catchment zone that comprises a certain population depending on the size of the catchment zone and population density. This concept implies that the covered population is specific to the respective city or urban area and depends on the definition of the catchment zone and dispatch criteria. The aim of efficient dispatching is to maximize the coverage of ischemic strokes and to minimize dispatches to patients with stroke mimics. This implies application of locally adapted MSU-specific alarming strategies.

Multiple factors limit the maximum number of stroke cases admitted to MSU: Cases in which EMS is not part of the rescue chain, for instance in patients who present themselves at the hospital, or when ischemic stroke is not being suspected at dispatch. Moreover, when employment of MSU would cause a significant delay or in case of other logistic impediments, the MSU could not reach stroke patients, which is the case particularly in peak hours and in large catchment zone sizes with long driving distances.

For this analysis, conservative assumptions were made to prevent possible bias leading to an overestimation of MSU performance. Based on data of Fladt et al originating from Basel, Switzerland, we assumed that 62% of all ischemic stroke patients were handled by EMS^2^. The remaining 38% of patients were assumed to directly present at the hospital or outpatient services or to suffer strokes while in the hospital. Furthermore, patients not identified at dispatch (false-negatives) have been described to range from around 23-51% of stroke patients^3,6–10^. As circadian stroke distribution is known to reach its peak around lunchtime^4^, and the probability of simultaneous events is increased in larger catchment zone populations, it was assumed that the proportion of missed stroke cases increased for larger catchment zones. However, detailed data on this magnitude are scarce. Based on a thorough review of the literature^5^, an additional rate of 2.5% missed cases was assumed per 100.000 inhabitants in the catchment zone.

The rate of missed strokes was simulated as follows:

38% (Stroke patients who did not come into contact with EMS) + 37% (missed strokes due to misidentification at dispatch) +8% (caused by logistic problems) + 2.5% / 100,000 population covered (higher frequency of simultaneous events and longer driving distances for larger populations, as well as saturation effects).

### Estimation of stroke-induced long-term inpatient healthcare costs

Based on the findings of Matchar et al ^16^, which are based on data originating from the Survey of Health, Ageing and Retirement in Europe (SHARE) survey, yearly stroke-induced additional hospitalization days for stationary care of stroke survivors were estimated according to category of functional impairment (none / moderate / severe) were calculated.

The unit costs for one day of hospitalization were extracted from Luengo-Fernandez et al ^17^, which was based on official healthcare cost data from Germany and inflated to 2021 €.

| **ADL impact** | **mRS** | **Additional Hospital days (annually)** | **Additional costs (annually)** |
| --- | --- | --- | --- |
| none | 0 | 0 | 0 |
| none | 1 | 0 | 0 |
| moderate | 2 | 5,4 | 4,838 |
| moderate | 3 | 5,4 | 4,838 |
| severe | 4 | 17,4 | 15,590 |
| severe | 5 | 17,4 | 15,590 |

**eTable 4: Stroke-induced inpatient healthcare costs according to functional status, which was defined based on activities of daily living (ADL) and mRS (Matchar et al).**

As there was no mRS-level adjusted data available on the number of additional general practitioner (GP) visits, this category of costs was not included.

### Estimation of long-term stroke-induced nursing costs

In order to estimate the long-term costs of stroke survivors from the perspective of the German social system, the costs of care for disability according to a specific level of disability to the public were considered.

In Germany, insurance-provided financial support for care is being provided according to the system of care degrees (CDs). To estimate potential costs, post-stroke mRS states were converted to average CDs according to multiple expert interviews conducted for this analysis, an online CD calculator was used to match the disability to a specific CD ^18^. Moreover, according to the analysis of Diederichs et al, only a limited proportion of patients requests financial support, differing for each mRS level ^19^. Based on official German statistics, a specific percentage of each CD group was estimated to receive care at home by the family, to receive care at home by family members together with a nursing service, or to receive care in a nursery home ^20^.

| **mRS Score** | **CD group (Pflegegrad)** | **Proportion of patients requesting financial aid** | **Home care delivered by family** | **Home care delivered by nursing service** | **Nursery home** |
| --- | --- | --- | --- | --- | --- |
| 0 | 0 | - | - | - | - |
| 1 | 0 | - | - | - | - |
| 2 | CD 1 | 24,50% | 85,00% | 14,20% | 0,80% |
| 3 | CD 3 | 33,30% | 57,16% | 22,76% | 20,08% |
| 4 | CD 4 | 63,30% | 40,54% | 20,61% | 38,85% |
| 5 | CD 5 | 76,50% | 31,98% | 19,39% | 48,64% |

**eTable 5: Estimation of long-term care needs based on mRS scores.**

The annual long-term public financial support according to each category of care was extracted from an official 2023 publication of the German Federal Ministry of Health ^21^.

| **mRS Score** | **Family-provided home based care** | **Contributions in kind for nursery service** | **Full inpatient care** |
| --- | --- | --- | --- |
| 0 | 0 € | 0 € | 0 € |
| 1 | 0 € | 0 € | 125 € |
| 2 | 316 € | 724 € | 770 € |
| 3 | 545 € | 1,363 € | 1,262 € |
| 4 | 728 € | 1,693 € | 1,775 € |
| 5 | 901 € | 2,095 € | 2,005 € |

**eTable 6: Financial support provided by German statutory nursing care insurance**

Additionally, for the patients that require long-term care in inpatient nursery services, the patient / the family typically covers additional nursery costs (called Einrichtungseinheitlicher Eigenanteil, Investitionskosten, Unterkunft und Verpflegung). Based on official data from the vdek - Verband der Ersatzkassen - ^22^, an average of these costs was calculated, deflated to 2021 € (resulting in 1760 € monthly costs ) and selectively added to the patients that require stationary nursing

The resulting final average costs of long-term care from the German societal perspective stratified for levels of disability (mRS states) are displayed in eTable 7.

| **mRS Score** | **Average annual costs** |
| --- | --- |
| 0 | 0 € |
| 1 | 0 € |
| 2 | 44 € |
| 3 | 4,909 € |
| 4 | 15,324 € |
| 5 | 23,186 € |

**eTable 7: Average costs of long-term nursing stratified for mRS states.**

### Estimation of long-term stroke-induced productivity losses

To account for annual lost productivity, the human capital approach was used. Age-adjusted percentage of population employed was extracted from data from the Genesis-online database by Statistisches Bundesamt (code 12211-0001, Bevölkerung, Erwerbstätige, Erwerbslose, Erwerbspersonen, Nichterwerbspersonen aus Hauptwohnsitzhaushalten: Deutschland, Jahre, Geschlecht, Altersgruppen) ^23^, productivity losses caused by stroke were considered according to data from Vyas et al 2016 ^24^. Chance to return to work after stroke was considered depending on mRS state ^25^. Average annual earnings (€ 49,260) in Germany were estimated based on official publications by the Statistisches Bundesamt ^26^. For patients that die, the average age-adjusted rest-productivity in € was included as additional costs.

### Long-term informal care needs

Stroke survivors are known to require additional informal care for example by the family according to level of disability ^16^. As the official insurance system in Germany partly reimburses costs, these were not additionally included to prevent possible bias which could overstate MSU cost-effectiveness.

## Economic modeling input parameters

The detailed set of input parameters that have not been described before and that were applied for economic modeling are fully disclosed in eTable 8. All costs were inflated to 2021 € based on the general German consumer price index (CPI).

| **Parameter** | **Value** | **Distribution in probabilistic sensitivity analysis** | **Source** |
| --- | --- | --- | --- |
| **Initial probabilities and assumptions** | | | |
| Mean age | 72.5 years | - | Average from German MSU trials of Kunz et al, Ebinger et al, Rohmann et al, Walter et al ^11,27–29^ |
| Discount rate | 3% | - | Attema et al 2018 ^30^ |
| Willingness-to-pay threshold per QALY | WHO Choice Highly cost-effective treshold (1x GDP per capita):  € 47,901 | - | WHO threshold: McDougall et al 2020  and  Data from the World Bank 2021 ^31,32^ |
| Model time horizon | 5 Years | - |  |
| Population in catchment area (base case) | 500,000 | normal | Estimate of realistic average catchment zone in Germany |
| **Transition probabilities** | | | |
| Hazard ratio for annual excess mortality following stroke (mRS 0-5) | 1.53 /  1.52 /  2.17 /  3.18 /  4.55 /  6.55 | lognormal | Hong et al 2010 ^33^ |
| Probability of stroke recurrence (year 1-10) | 5.9% /  3.6% /  2.5% /  2.2% /  2.2% /  2.7% /  2.7% /  2.3% /  2.8% /  1.6% | beta | Pennlert et al 2014 ^34^ |
| Age-adjusted mortality rates | German mortality rates 2019 | beta | Derived from official German population statistics by Statistisches Bundesamt (Destatis), 2019 ^35^ |
| **Healthcare costs** | | | |
| MSU investment costs, depreciated to five years, average | € 169,361 | gamma | Averages from Kim et al 2021 ^36^, Gyrd-Hansen et al 2015 ^37^ and Dietrich et al 2014 ^38^ |
| MSU running costs per year | € 150,790 | gamma |  |
| Staffing costs according to staffing model | See eTable 3 | gamma | Calculation based on cost data from hospital accounting and local EMS accounting department, based on official tariffs |
| Direct costs of acute stroke care, initial hospital admission | mRS 0-2:  € 4,492  mRS 3-6:  € 5,052 | gamma | Dodel et al 2004 ^39^ |
| Long-term healthcare costs due to stroke-induced hospitalisations stratified for mRS levels  (0/ 1/ 2/ 3/ 4/ 5) | see eTable 4 | gamma | Disability-adjusted resource use based on Matchar et al 2015 ^16^ and costs based on Luengo-Fernandez et al ^17^ |
| Costs of IVT | € 1,468 | gamma | Muntendorf et al 2021 ^40^ |
| Costs of transfer (EMS and emergency physician), applied to transfer of ICH and stroke patients | € 923 | gamma | Averages from Berlin and Hemer ^41^ |
| Costs of stroke mimics in emergency department (exemplary for epilepsia, first episode) | € 2,372 | gamma | Strzelczyk et al 2013 ^42^ |
| Costs of ICH treatment, direct costs for one year | € 26,646 | gamma | Rossnagel et al 2005 ^43^ |
| Costs of TIA treatment, direct costs for one year | € 11,100 | gamma | Rossnagel et al 2005 ^43^ |
| **Societal costs / transfer payments** | | | |
| Percentage of population employed according to age | Official German employment rates | beta | Data from Statistisches Bundesamt, Genesis Database code 12211-0001 ^23^ |
| Productivity loss caused by stroke | 17.5% | beta | Vyas et al 2016 ^24^ |
| Probability of returning to work after stroke according to mRS state | 62.5% / 71.7% /  49.2% /  18.8% /  13.6% /  0% | beta | Tanaka et al 2014 ^44^ |
| Average individual annual productivity in Germany | € 49,260 | gamma | Statistisches Bundesamt ^26^ |
| Transfer payments for long-term nursing care stratified to mRS levels  (0/ 1/ 2/ 3/ 4/ 5) | see eTable 7 | gamma | Conversion of mRS-dependent long-term disability to German CDs (Pflegegrade) and associated costs ^19,21^, further details see above |
| **Outcomes and utility levels** | | | |
| Clinical outcomes of stroke patients after 90 days: Proportion of patients in each mRS level (0/ 1/ 2/ 3/ 4/ 5) | MSU:  24.9% / 19.7% / 12.3% / 17.1% / 13.8% / 6.6% /  5.5%  EMS:  18.4% / 18.4% / 15.0% / 18.6% / 14.4% / 7.6% / 7.6% | Dirichlet | Patient-number adjusted averages from patients with ischemic stroke without mimics, without hemorrhages,  Grotta (n=1103) ^12^ and  Ebinger (n=1337) ^11^ |
| Clinical outcomes of TIA patients, mRS levels 0/ 1/ 2/ 3/ 4/ 5 | 21.7% / 19.1% / 13.7% / 17.9% / 14.1% / 7.1% / 6.6% | Dirichlet | Outcomes were assumed not to be changed by MSU, based on Grotta et al 2021 ^12^ and  Ebinger (n=1337) ^11^ |
| Clinical outcomes of ICH and mimics patients (no improvement of outcomes due to MSU deployment assumed), mRS levels 0/ 1/ 2/ 3/ 4/ 5 | 9.4% / 14.6% / 10.1% / 13,0% / 25.9% / 19.9% / 7.2% | Dirichlet | Estimate, based on Grotta et al 2021 ^12^ - outcome unchanged by MSU. |
| Utilities for Germany, mRS levels 0/ 1/ 2/ 3/ 4/ 5 | 0.95 / 0.90 / 0.83 / 0.68 / 0.38 / 0.09 | beta | Ali et al 2015 ^45^ |

**eTable 8: Model input parameters**

**Validation of newly-introduced critical input parameters**

| **Parameter** | **Validated by** |
| --- | --- |
| Stroke prevalence, incidence | Acute stroke incidence in the City of Mannheim - 4,93% difference to the input parameter used |
| Probability of stroke patients not alarming EMS | Confirmation with hospital Stroke Unit quality management data |
| Probability of alternative diagnoses within MSU | Expert confirmation by Audebert HJ (Co-author) |
| Percentages of stroke admissions occurring during different time windows | Confirmation with clinical data from our institution: 7,79% difference in stroke admissions within 8h admission window, 4,73% difference within 16h window. |
| Probability of misidentification of stroke by dispatch center | Confirmation with local dispatch center (Integrierte Rettungsleitstelle Mannheim), using preliminary data from an ongoing study. |
| Proportion of MSU-admitted patients receiving full MSU management | Expert confirmation by Audebert HJ (Co-author) |
| Unit costs for 16h/7day model | Unit costs are dependent on hardware depreciation time frames, therefore limited comparability.  In comparison with preliminary costing data from the anticipated Mannheim Mobile Stroke Unit, the 16h/7days model differs 3,56% in annual hardware and running costs and 1,78% in personnel costs. |
| Personnel costs for physicians / radiology technician / paramedic | Data originating from hospital and EMS (Deutsches Rotes Kreuz Mannheim e.V.) Accounting Departments. |
| Long-term mRS-stratified post-stroke care unit costs/transfer payments for hospitalization, nursing and productivity losses | N/A - as this was the first analysis that used the described methodology.  Analyses from Gonçalves et al and Muntendorf et al both used different methodologies. |
| Outcomes in Ischemic stroke patients | Comparison with outcomes from other MSU studies based on the review from Chen et al ^46^ |
| Modeled long-term survival of stroke patients | See eFigure 5 and eTable 12 |

**eTable 9: Model input parameter validation**

Newly introduced, critical input parameters were validated as shown in eTable 9. Other parameters, for example the transition probabilities extracted from literature, have been utilized for stroke-specific cost-effectiveness analyses over years.

### Results: Operation modes, coverage, catchment zones and corresponding ICERs

The effect of varying operating modes on coverage of ischemic strokes and corresponding measures of cost-effectiveness (ICER) is provided in eTable 6.

| **Operation mode** | **ICER (societal perspective)** | **ICER (healthcare perspective)** | **Patient-level short-term costs** | **Coverage** |
| --- | --- | --- | --- | --- |
| **8h5d** | € 18,927 | € 26,849 | € 8,046 | 16.8% |
| **8h6d** | € 20,327 | € 28,249 | € 8,383 | 20.1% |
| **8h7d** | € 17,634 | € 25,556 | € 7,734 | 23.5% |
| **12h5d** | € 15,208 | € 23,131 | € 7,149 | 23.3% |
| **12h6d** | € 15,454 | € 23,376 | € 7,208 | 28.0% |
| **12h7d** | € 14,174 | € 22,096 | € 6,899 | 32.6% |
| **16h5d** | € 15,142 | € 23,064 | € 7,133 | 28.3% |
| **16h6d** | € 12,138 | € 20,060 | € 6,408 | 33.9% |
| **16h7d** | € 9,922 | € 17,843 | € 5,873 | 39.6% |
| **24h5d** | € 22,190 | € 30,112 | € 8,833 | 30.4% |
| **24h6d** | € 20,544 | € 28,466 | € 8,436 | 36.4% |
| **24h7d** | € 20,721 | € 28,643 | € 8,479 | 42.5% |

**eTable 10: MSU patient coverage and ICER for different coverage models, for a population of 500,000 inhabitants.**

| **Catchment zone population** | **Per-patient cost** | **ICER healthcare** | **ICER societal** |
| --- | --- | --- | --- |
| **200,000** | € 16,435 | € 73,642 | € 65,720 |
| **300,000** | € 13,485 | € 46,928 | € 39,006 |
| **400,000** | € 9,130 | € 33,571 | € 25,648 |
| **500,000** | € 7,734 | € 25,556 | € 17,634 |
| **600,000** | € 6,848 | € 20,213 | € 12,291 |
| **700,000** | € 6,261 | € 16,397 | € 8,475 |
| **800,000** | € 5,870 | € 13,535 | € 5,613 |
| **900,000** | € 5,619 | € 11,309 | € 3,387 |
| **1,000,000** | € 5,478 | € 9,528 | € 1,606 |

**eTable 11: Catchment zone population vs. Per-patient costs and ICERs**

**Sensitivity analysis - additional analyses**

**a)**

##
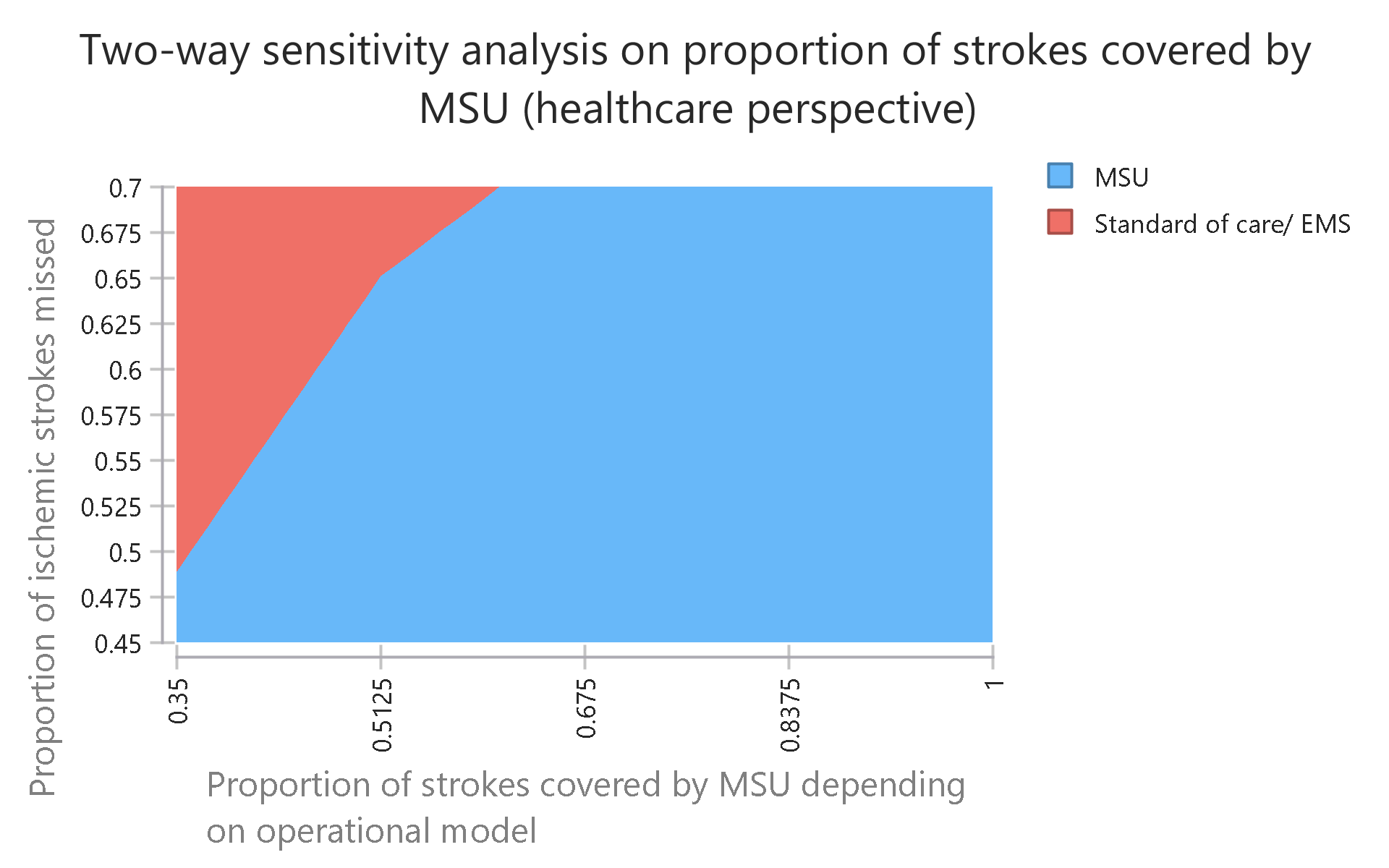


**b)**

##
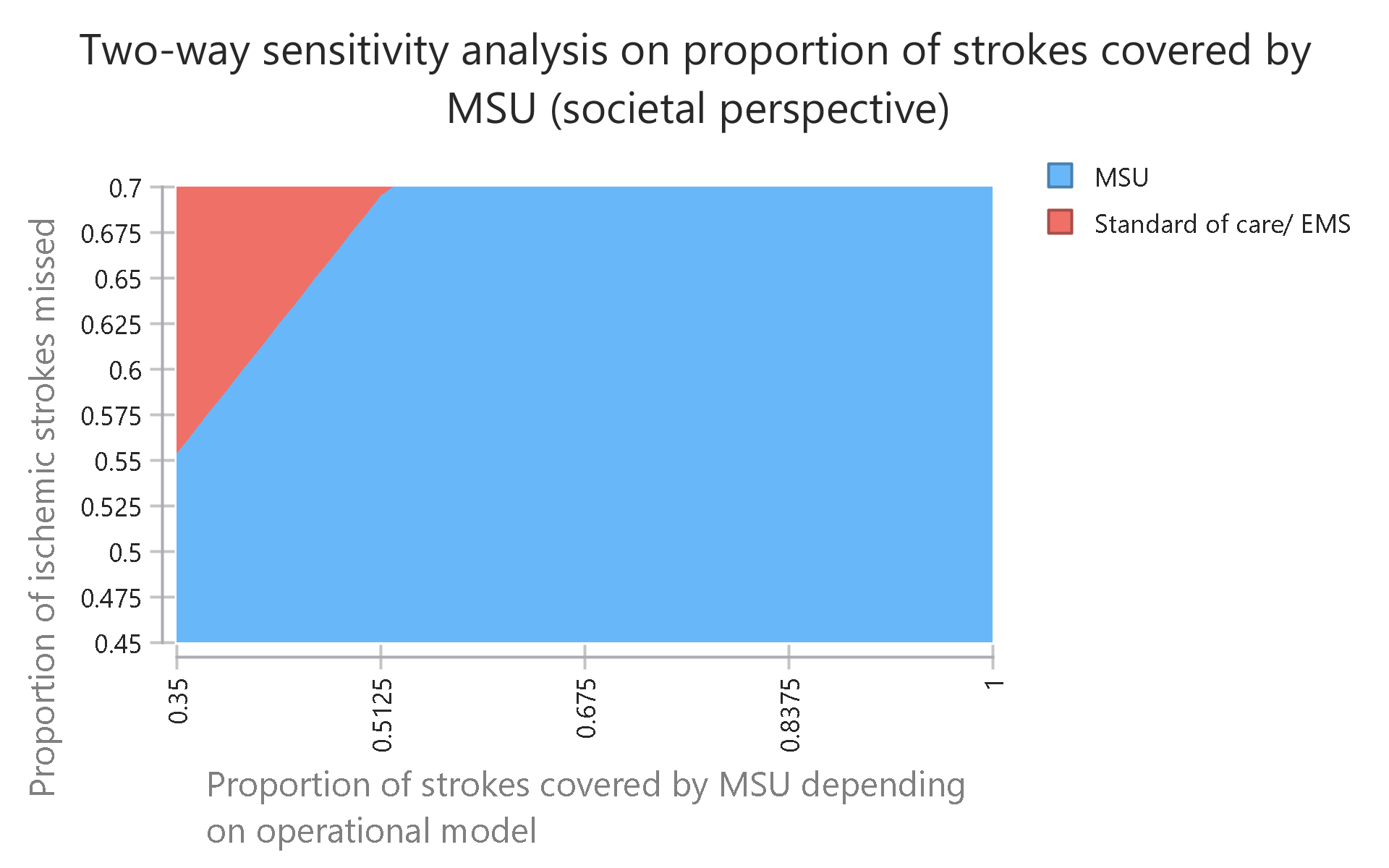


**eFigure 2: Two-way sensitivity analysis of the net monetary benefit of MSU**This analysis displays the relationship between net monetary benefit of the MSU strategy / standard of care strategy and the choice of operational model (8h5d - 24h7d) as well as the proportion of missed cases, for the healthcare perspective (a) and societal perspective (b). WTP was assumed at € 47,901 per QALY gained. Missing cases significantly deteriorated MSUs economic performance, and there are substantial economic differences between various operational modes.

## Adherence to established reporting standards

### CHEERS Checklist

The Consolidated Health Economic Evaluation Reporting Standards (CHEERS) Checklist 2022 was closely followed (eTable 7).

| **Element** | **Journal Article section / paragraph** | **Technical Appendix** |
| --- | --- | --- |
| **Introduction** |  |  |
| Background of the problem | Introduction / p1-3 |  |
| **Study Design and Scope** |  |  |
| Objectives | Introduction / p4 |  |
| Audience | Introduction / p1 |  |
| Type of analysis | Methods / p2 |  |
| Target populations | Methods / p2,6 |  |
| Description of interventions and comparators (including no intervention, if applicable) | Methods / p2,3,5 |  |
| Other intervention descriptors (eg, care setting, model of delivery, intensity and timing of intervention) | Methods / p2-6 |  |
| Boundaries of the analysis; defining the scope or comprehensiveness of the study (eg, for a screening program, whether only a subset of many possible strategies are included; for a transmissible condition, the extent to which disease transmission is captured;  for interventions with many possible delivery settings, whether only one or more settings are modeled) | Methods / p2,11-13 |  |
| Time horizon | Methods / p2 |  |
| Analytic perspectives (eg, reference case perspectives [health care sector, societal]; other perspectives such as employer or payer) | Methods / p7 |  |
| Whether this analysis meets the requirements of the reference case | Methods / - |  |
| Analysis plan | Methods / p5 |  |
| **Methods and Data** |  |  |
| Description of event pathway or model (describe condition or disease and the health states included) | Methods / p2-5 | e-Figure 1 + legend |
| Diagram of event pathway or model (depicting the sequencing and possible transitions among the health states included) | Figure 1 + legend |  |
| Description of model used (eg, decision tree, state transition, microsimulation) | Methods / p2 |  |
| Modeling assumptions | Methods / p3, 6, 7, 10, 13 | eTable 1-8 |
| Software used | Methods / p2 |  |
| Identification of key outcomes | Methods / p5 |  |
| Complete information on sources of effectiveness data, cost data, and preference weights | Methods / p3, 6-9 | eTable 1-8 |
| Methods for obtaining estimates of effectiveness (including approaches used for evidence synthesis) | Methods / p9 |  |
| Methods for obtaining estimates of costs and preference weights | Methods / p7,8 |  |
| Critique of data quality | Discussion / p8 |  |
| Statement of costing year (ie, the year to which all costs have been adjusted for the analysis; eg, xxxx) | Methods / p7 |  |
| Statement of method used to adjust costs for inflation | Methods / p7 |  |
| Statement of type of currency | Methods / p7 |  |
| Source and methods for obtaining expert judgment if applicable | N/A |  |
| Statement of discount rates | Methods / p5 |  |
| **Impact Inventory** |  |  |
| Full accounting of consequences within and outside the health care sector | Discussion / p9 |  |
| **Results** |  |  |
| Results of model validation |  | e-Figure 5,  e-Table 9 |
| Reference case results (discounted and undiscounted): total costs and effectiveness, incremental costs and effectiveness, incremental cost-effectiveness ratios, measures of uncertainty | Results / p2 Table 1 | e-Table 10,11 |
| Disaggregated results for important categories of costs, outcomes, or both | Table 1 |  |
| Results of sensitivity analysis | Results p 3-6, Figure 2-4 | e-Figure 2 |
| Other estimates of uncertainty | N/A |  |
| Graphical representation of cost-effectiveness results | Figures 2,3 |  |
| Graphical representation of uncertainty analyses | Figure 4 | e-Figure 2 |
| Aggregate cost and effectiveness information | Results p2 |  |
| Secondary analyses | Results p3,4, Figure 2/3 |  |
| **Disclosures** |  |  |
| Statement of any potential conflicts of interest due to funding source, collaborations, or outside interests | see Disclosures |  |
| **Discussion** |  |  |
| Summary of reference case results | Discussion / p2 |  |
| Summary of sensitivity of results to assumptions and uncertainties in the analysis | Discussion / p8 |  |
| Discussion of the study results in the context of results of related cost-effective analyses | Discussion / p6 |  |
| Discussion of ethical implications (eg, distributive implications relating to age, disability, or other characteristics of the population) | N/A |  |
| Limitations of the study | Discussion / p8 |  |
| Relevance of study results to specific policy questions or decisions | Discussion / p9 |  |

**eTable 12: CHEERS checklist**

## Size of catchment zones reported by international MSU projects and association of driving distance and catchment zone population in Mannheim

Literature research revealed a very broad range of reported response areas by multiple MSU projects worldwide ^5,38,47–54^. Some projects reported the covered population of the response area (ranging from around 180,000 to > 2,000,000) whereas others reported driving distances of MSU around a central base station (from around 8 miles to 155 miles). As most projects operate under urban conditions, average driving distances are relatively short, however operating ranges can be extended by a rendezvous system. Rural MSU services require much higher distances to cover patients.


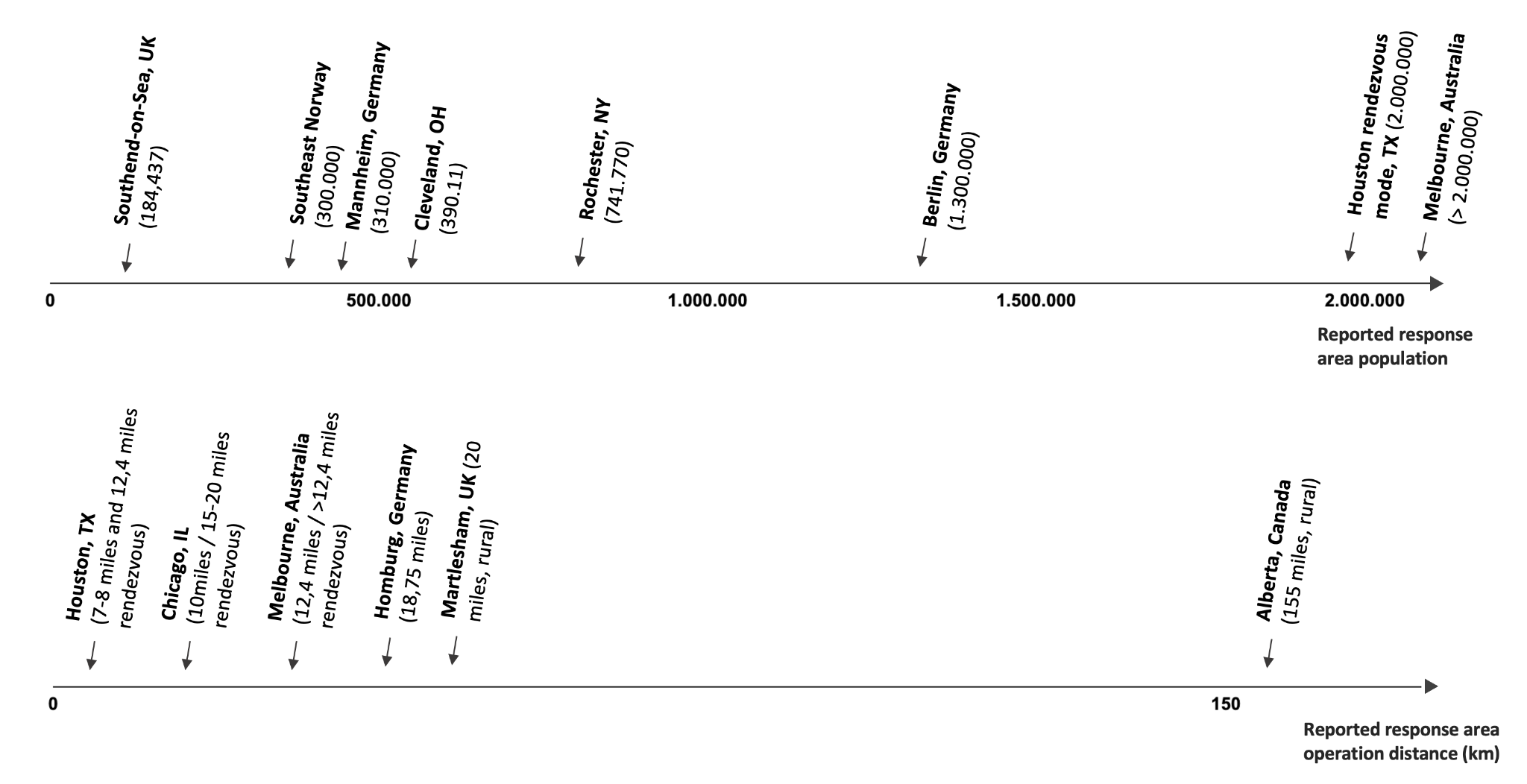
**eFigure 3: Range of reported response area populations and operation distances of MSU projects**

The impact of extending the catchment zone is demonstrated in eFigure 3 for the German city of Mannheim. When the MSU service covered only the inner city, only 310,000 inhabitants could be covered and maximum driving times of 15.4 minutes could be achieved. By assuming a rendezvous system, over 1 million inhabitants could be covered, which substantially reduced the costs per patient.


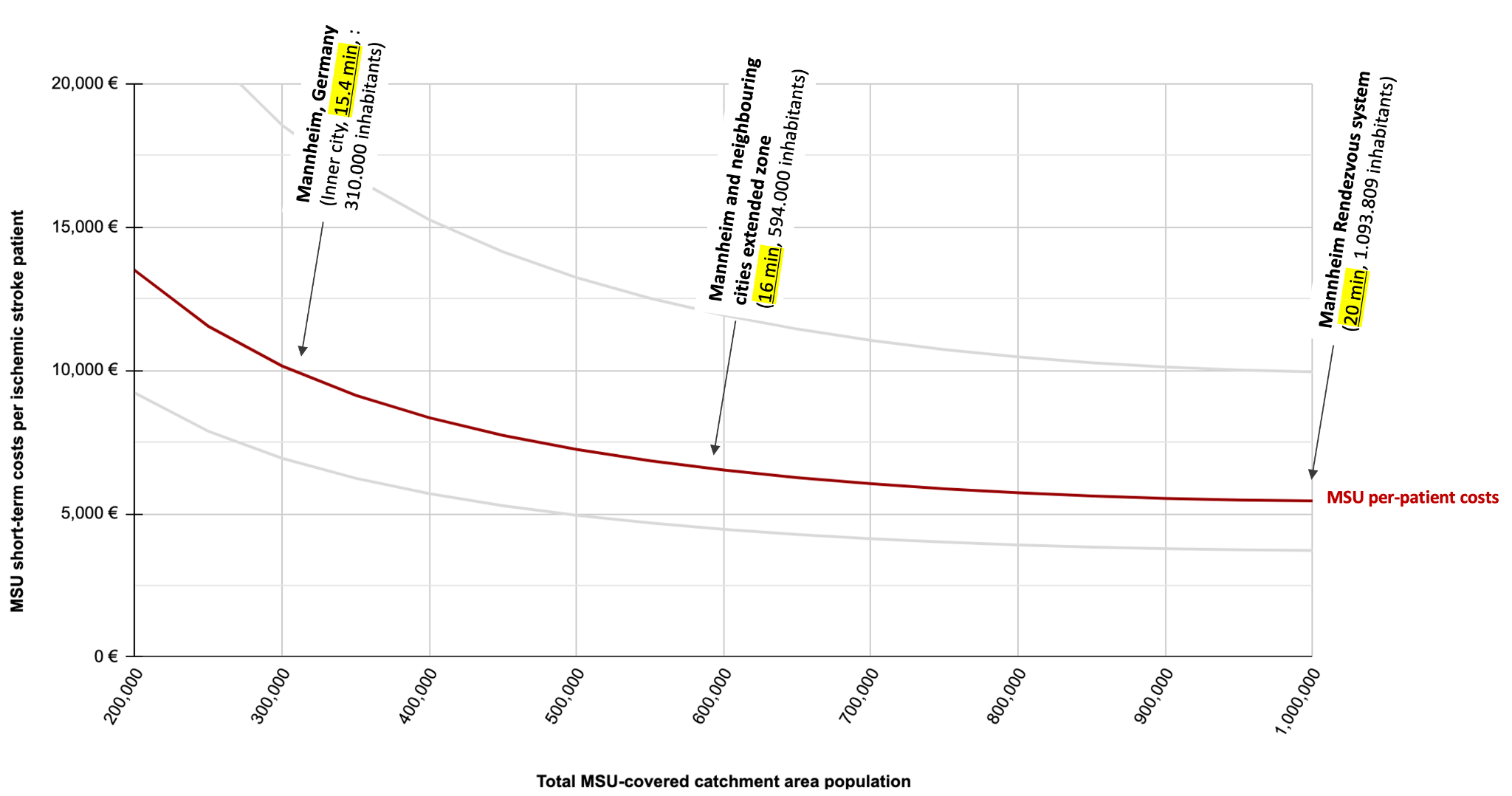


**eFigure 4: Exemplary impact of driving radius on catchment zone population in Mannheim**

Inclusion of neighboring cities and increase of driving radius around the central base substantially increases the included population of the catchment zone.

##

## Comparison of outcomes with other MSU cost-effectiveness analyses

Our modeling results were compared with main outcomes of other cost-effectiveness analyses of Mobile Stroke Unit services in eTable 8.

| **Study** | **Number of treated ischemic stroke patients** | **Perspective** | **Included long-term costs** | **Incremental short-term MSU costs** | **Incremental long-term MSU costs** | **Incremental**  **long-term QALYs per patient** | **ICER** |
| --- | --- | --- | --- | --- | --- | --- | --- |
| Gonçalves et al (Berlin) ^55^  (real-world) | 1,543 in 826 days (681 patients annually, three MSUs) | 5 year, societal (and additional other) perspectives | Nursing costs by social insurance | € 7,217 | € 6,973 | 0.17  (262.52 for 1,543 patients) | € 40,984  per QALY (societal perspective) |
| Lund et al (Norway) ^56^  (model) | 180 annually in main scenario | lifetime, national health services | Swedish long-term costs data | - | USD 4,157 | 0.065 | USD 63,670 per QALY |
| This analysis  (model) | 116 annually (Catchment zone of 500,000 inhabitants and 8h/7d coverage) | 5 year German societal and healthcare perspective | Nursing costs,  Hospital readmissions,  productivity losses,  private additional costs for nursing | € 2,156  (including TIA and stroke mimics) | € 1,444 (healthcare)€ 996 (societal)  (including TIA and stroke mimics | 0.06 | € 17,634 per QALY (societal perspective)  € 25,556 (healthcare perspective) |

**eTable 13: Comparison: Economic evaluations on MSU services**

###

### Validation of modeled survival rates

To confirm model accuracy and prevent any modeling errors compromising the validity of the results, survival rates of the patients stratified for mRS categories were modeled and compared to the published post-stroke survival rates of the UK Lothian cohort ^57^. Overall, there was sufficient matching between model survival and reported real-world survival rates (eFigure 4).


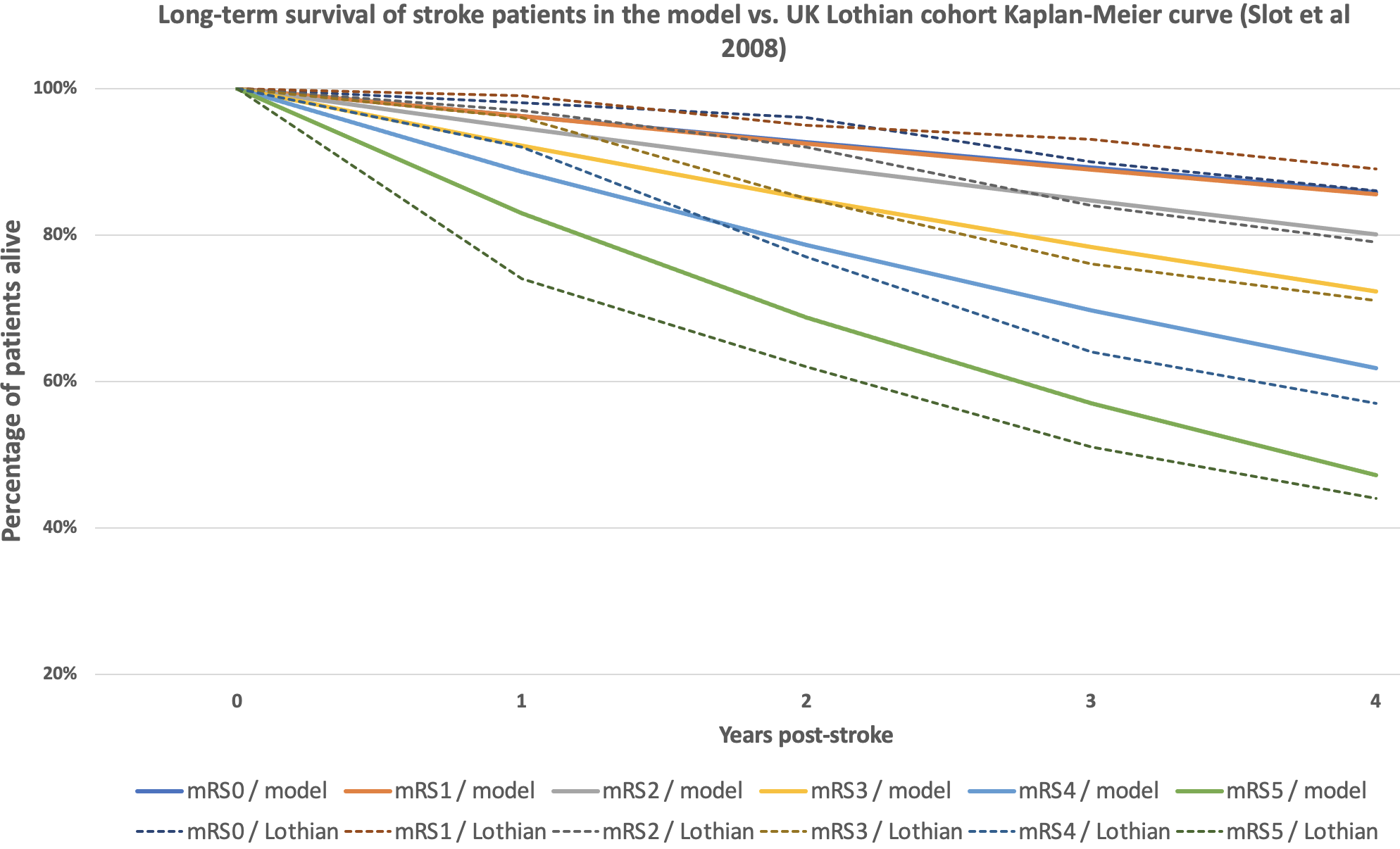


**eFigure 5: Survival according to mRS states**

| **Year** | **0** | **1** | **2** | **3** | **4** |
| --- | --- | --- | --- | --- | --- |
| **mRS0** | 1 | 0.962 | 0.926 | 0.892 | 0.858 |
| **mRS1** | 1 | 0.962 | 0.925 | 0.889 | 0.855 |
| **mRS2** | 1 | 0.946 | 0.895 | 0.847 | 0.801 |
| **mRS3** | 1 | 0.922 | 0.850 | 0.784 | 0.723 |
| **mRS4** | 1 | 0.887 | 0.786 | 0.697 | 0.618 |
| **mRS5** | 1 | 0.829 | 0.687 | 0.570 | 0.472 |

**eTable 12: Modeled survival rates stratified to mRS states**

##

## Supplemental References

1. Gesundheitsberichterstattung des Bundes. Daten zu Schlaganfällen in der Stadt Erlangen 2021. Accessed December 27, 2022. https://www.gbe-bund.de/gbe/ergebnisse.prc_tab?fid=8299&suchstring=&query_id=&sprache=D&fund_typ=TAB&methode=&vt=&verwandte=1&page_ret=0&seite=1&p_lfd_nr=2&p_news=&p_sprachkz=D&p_uid=gast&p_aid=70345628&hlp_nr=2&p_janein=J

2. Fladt J, Meier N, Thilemann S, et al. Reasons for Prehospital Delay in Acute Ischemic Stroke. *Journal of the American Heart Association*. 2019;8(20):e013101. doi:10.1161/JAHA.119.013101

3. Fassbender K, Phillips DJ, Grunwald IQ, et al. Hybrid‐Mobile Stroke Unit: Opening the Indication Spectrum for Stroke Mimics and Beyond. *Stroke: Vascular and Interventional Neurology*. 2023;3(1):e000482. doi:10.1161/SVIN.122.000482

4. Wroe SJ, Sandercock P, Bamford J, Dennis M, Slattery J, Warlow C. Diurnal variation in incidence of stroke: Oxfordshire community stroke project. *BMJ*. 1992;304(6820):155-157.

5. Ellens NR, Schartz D, Rahmani R, et al. Mobile Stroke Unit Operational Metrics: Institutional Experience, Systematic Review and Meta-Analysis. *Frontiers in Neurology*. 2022;13. Accessed December 16, 2022. https://www.frontiersin.org/articles/10.3389/fneur.2022.868051

6. Mattila OS, Puolakka T, Ritvonen J, et al. Targets for improving dispatcher identification of acute stroke. *Int J Stroke*. 2019;14(4):409-416. doi:10.1177/1747493019830315

7. Mould-Millman NK, Meese H, Alattas I, et al. Accuracy of Prehospital Identification of Stroke in a Large Stroke Belt Municipality. *Prehosp Emerg Care*. 2018;22(6):734-742. doi:10.1080/10903127.2018.1447620

8. Nour M, Kazan C, Steeneken N, et al. Abstract 35: Dispatcher Impression of Stroke: Concordance With Paramedic Identification and Effect on Allocation of Standard Ambulances and Mobile Stroke Units. *Stroke*. 51(Suppl_1):A35-A35. doi:10.1161/str.51.suppl_1.35

9. Eliakundu AL, Cadilhac DA, Kim J, et al. Determining the sensitivity of emergency dispatcher and paramedic diagnosis of stroke: statewide registry linkage study. *J Am Coll Emerg Physicians Open*. 2022;3(4):e12750. doi:10.1002/emp2.12750

10. Bohm K, Kurland L. The accuracy of medical dispatch - a systematic review. *Scand J Trauma Resusc Emerg Med*. 2018;26(1):94. doi:10.1186/s13049-018-0528-8

11. Ebinger M, Siegerink B, Kunz A, et al. Association Between Dispatch of Mobile Stroke Units and Functional Outcomes Among Patients With Acute Ischemic Stroke in Berlin. *JAMA*. 2021;325(5):454-466. doi:10.1001/jama.2020.26345

12. Grotta JC, Yamal JM, Parker SA, et al. Prospective, Multicenter, Controlled Trial of Mobile Stroke Units. *N Engl J Med*. 2021;385(11):971-981. doi:10.1056/NEJMoa2103879

13. Helwig SA, Ragoschke-Schumm A, Schwindling L, et al. Prehospital Stroke Management Optimized by Use of Clinical Scoring vs Mobile Stroke Unit for Triage of Patients With Stroke: A Randomized Clinical Trial. *JAMA Neurol*. 2019;76(12):1484-1492. doi:10.1001/jamaneurol.2019.2829

14. Backhaus R, Schlachetzki F, Rackl W, et al. Intracranial hemorrhage: frequency, location, and risk factors identified in a TeleStroke network. *Neuroreport*. 2015;26(2):81-87. doi:10.1097/WNR.0000000000000304

15. DFG-Programmpauschale - BMBF. Bundesministerium für Bildung und Forschung - BMBF. Accessed March 27, 2023. https://www.bmbf.de/bmbf/de/forschung/das-wissenschaftssystem/dfg-programmpauschale/dfg-programmpauschale_node.html

16. Matchar DB, Bilger M, Do YK, Eom K. International Comparison of Poststroke Resource Use: A Longitudinal Analysis in Europe. *Journal of Stroke and Cerebrovascular Diseases*. 2015;24(10):2256-2262. doi:10.1016/j.jstrokecerebrovasdis.2015.06.020

17. Luengo-Fernandez R, Violato M, Candio P, Leal J. Economic burden of stroke across Europe: A population-based cost analysis. *European Stroke Journal*. 2020;5(1):17-25. doi:10.1177/2396987319883160

18. e.V SVD. Pflegebedürftig? - Hier finden Sie Tipps und wichtige Informationen. Accessed April 18, 2023. https://www.vdk.de/deutschland/pages/themen/pflege/74125/pflegebeduerftig_tipps_pflegebegutachtung_pflegegradrechner_kostenlos

19. Ärzteblatt DÄG Redaktion Deutsches. Prädiktoren für eine spätere Pflegebedürftigkeit nach einem Schlaganfall. Deutsches Ärzteblatt. Published September 9, 2011. Accessed March 6, 2023. https://www.aerzteblatt.de/archiv/104400/Praediktoren-fuer-eine-spaetere-Pflegebeduerftigkeit-nach-einem-Schlaganfall

20. Pflegebedürftige nach Versorgungsart, Geschlecht und Pflegegrade. Statistisches Bundesamt. Accessed June 18, 2023. https://www.destatis.de/DE/Themen/Gesellschaft-Umwelt/Gesundheit/Pflege/Tabellen/pflegebeduerftige-pflegestufe.html

21. Pflegeleistungen zum Nachschlagen. Accessed April 18, 2023. https://www.bundesgesundheitsministerium.de/service/publikationen/details/pflegeleistungen-zum-nachschlagen.html

22. Finanzielle Belastung der Pflegebedürftigen in Pflegeheimen steigt kontinuierlich. Accessed January 14, 2024. https://www.vdek.com/presse/pressemitteilungen/2023/pflegeheim-finanzielle-belastung-steigt-kontinuierlich.html

23. Statistisches Bundesamt Deutschland - GENESIS-Online. Published January 2, 2024. Accessed January 2, 2024. https://www-genesis.destatis.de/genesis//online?operation=table&code=12211-0001&bypass=true&levelindex=0&levelid=1704187171536#abreadcrumb

24. Vyas MV, Hackam DG, Silver FL, Laporte A, Kapral MK. Lost Productivity in Stroke Survivors: An Econometrics Analysis. *Neuroepidemiology*. 2016;47(3-4):164-170. doi:10.1159/000454730

25. Tanaka K, Reeves MJ. Mobile Stroke Units and Pursuit of Intravenous Tissue-Type Plasminogen Activator Treatment in the Golden Hour. *Stroke*. 2023;54(2):426-429. doi:10.1161/STROKEAHA.122.041391

26. Verdienste 2021: Durchschnittlich 4 100 Euro brutto im Monat. Statistisches Bundesamt. Accessed January 2, 2024. https://www.destatis.de/DE/Themen/Arbeit/Verdienste/Verdienste-Branche-Berufe/verdienste-branchen.html

27. Kunz A, Ebinger M, Geisler F, et al. Functional outcomes of pre-hospital thrombolysis in a mobile stroke treatment unit compared with conventional care: an observational registry study. *Lancet Neurol*. 2016;15(10):1035-1043. doi:10.1016/S1474-4422(16)30129-6

28. Walter S, Kostopoulos P, Haass A, et al. Diagnosis and treatment of patients with stroke in a mobile stroke unit versus in hospital: a randomised controlled trial. *Lancet Neurol*. 2012;11(5):397-404. doi:10.1016/S1474-4422(12)70057-1

29. Rohmann JL, Piccininni M, Ebinger M, et al. Effect of Mobile Stroke Unit Dispatch in all Patients with Acute Stroke or TIA. *Annals of Neurology*. n/a(n/a). doi:10.1002/ana.26541

30. Attema AE, Brouwer WBF, Claxton K. Discounting in Economic Evaluations. *Pharmacoeconomics*. 2018;36(7):745-758. doi:10.1007/s40273-018-0672-z

31. McDougall JA, Furnback WE, Wang BCM, Mahlich J. Understanding the global measurement of willingness to pay in health. *J Mark Access Health Policy*. 8(1):1717030. doi:10.1080/20016689.2020.1717030

32. World Bank Open Data. World Bank Open Data. Accessed April 18, 2023. https://data.worldbank.org

33. Hong KS, Saver JL. Years of disability-adjusted life gained as a result of thrombolytic therapy for acute ischemic stroke. *Stroke*. 2010;41(3):471-477. doi:10.1161/STROKEAHA.109.571083

34. Pennlert J, Eriksson M, Carlberg B, Wiklund PG. Long-term risk and predictors of recurrent stroke beyond the acute phase. *Stroke*. 2014;45(6):1839-1841. doi:10.1161/STROKEAHA.114.005060

35. Sterbefälle - Fallzahlen nach Tagen, Wochen, Monaten, Altersgruppen, Geschlecht und Bundesländern für Deutschland 2016 - 2023. Statistisches Bundesamt. Accessed April 18, 2023. https://www.destatis.de/DE/Themen/Gesellschaft-Umwelt/Bevoelkerung/Sterbefaelle-Lebenserwartung/Tabellen/sonderauswertung-sterbefaelle.html

36. Kim J, Easton D, Zhao H, et al. Economic evaluation of the Melbourne Mobile Stroke Unit. *Int J Stroke*. 2021;16(4):466-475. doi:10.1177/1747493020929944

37. Gyrd-Hansen D, Olsen KR, Bollweg K, Kronborg C, Ebinger M, Audebert HJ. Cost-effectiveness estimate of prehospital thrombolysis: results of the PHANTOM-S study. *Neurology*. 2015;84(11):1090-1097. doi:10.1212/WNL.0000000000001366

38. Dietrich M, Walter S, Ragoschke-Schumm A, et al. Is prehospital treatment of acute stroke too expensive? An economic evaluation based on the first trial. *Cerebrovasc Dis*. 2014;38(6):457-463. doi:10.1159/000371427

39. Dodel RC, Haacke C, Zamzow K, et al. Resource utilization and costs of stroke unit care in Germany. *Value Health*. 2004;7(2):144-152. doi:10.1111/j.1524-4733.2004.72314.x

40. Muntendorf LK, Konnopka A, König HH, et al. Cost-Effectiveness of Magnetic Resonance Imaging-Guided Thrombolysis for Patients With Stroke With Unknown Time of Onset. *Value Health*. 2021;24(11):1620-1627. doi:10.1016/j.jval.2021.05.005

41. VIS Berlin - Gebührenverzeichnis “B” - Besondere Benutzungen - Fw BenGebO | Landesnorm Berlin | Gebührenverzeichnis “B” - Besondere Benutzungen - | gültig ab: 11.09.2021. Accessed December 27, 2022. https://gesetze.berlin.de/bsbe/document/jlr-FeuerwEBenGebOBEV11Anl-G1

42. Strzelczyk A, Knake S, Oertel WH, Rosenow F, Hamer HM. Inpatient treatment costs of status epilepticus in adults in Germany. *Seizure - European Journal of Epilepsy*. 2013;22(10):882-885. doi:10.1016/j.seizure.2013.08.003

43. Rossnagel K, Nolte CH, Muller-Nordhorn J, et al. Medical resource use and costs of health care after acute stroke in Germany. *Eur J Neurol*. 2005;12(11):862-868. doi:10.1111/j.1468-1331.2005.01091.x

44. Tanaka H, Toyonaga T, Hashimoto H. Functional and occupational characteristics predictive of a return to work within 18 months after stroke in Japan: implications for rehabilitation. *Int Arch Occup Environ Health*. 2014;87(4):445-453. doi:10.1007/s00420-013-0883-8

45. Ali M, MacIsaac R, Quinn TJ, et al. Dependency and health utilities in stroke: Data to inform cost-effectiveness analyses. *European Stroke Journal*. 2017;2(1):70-76. doi:10.1177/2396987316683780

46. Chen J, Lin X, Cai Y, Huang R, Yang S, Zhang G. A Systematic Review of Mobile Stroke Unit Among Acute Stroke Patients: Time Metrics, Adverse Events, Functional Result and Cost-Effectiveness. *Front Neurol*. 2022;13:803162. doi:10.3389/fneur.2022.803162

47. Freitag E, Kaffes M, Weber JE, Audebert HJ. How to Set Up a Successfully Running Mobile Stroke Unit Program. *Stroke*. 2021;52(4):e107-e110. doi:10.1161/STROKEAHA.120.033576

48. Kerro A, Kus T, Cai C, et al. Abstract 32: “Rendezvous System” for Broadening a Mobile Stroke Unit Catchment Area. *Stroke*. 50(Suppl_1):A32-A32. doi:10.1161/str.50.suppl_1.32

49. Kate MP, Jeerakathil T, Buck BH, et al. Pre-hospital triage of suspected acute stroke patients in a mobile stroke unit in the rural Alberta. *Sci Rep*. 2021;11(1):4988. doi:10.1038/s41598-021-84441-0

50. Kummer BR, Lerario MP, Hunter MD, et al. Geographic Analysis of Mobile Stroke Unit Treatment in a Dense Urban Area: The New York City METRONOME Registry. *Journal of the American Heart Association*. 2019;8(24):e013529. doi:10.1161/JAHA.119.013529

51. Larsen K, Jaeger HS, Tveit LH, et al. Ultraearly thrombolysis by an anesthesiologist in a mobile stroke unit: A prospective, controlled intervention study. *Eur J Neurol*. 2021;28(8):2488-2496. doi:10.1111/ene.14877

52. Weinberg JH, Sweid A, DePrince M, et al. The impact of the implementation of a mobile stroke unit on a stroke cohort. *Clin Neurol Neurosurg*. 2020;198:106155. doi:10.1016/j.clineuro.2020.106155

53. Zhao H, Coote S, Easton D, et al. Melbourne Mobile Stroke Unit and Reperfusion Therapy: Greater Clinical Impact of Thrombectomy Than Thrombolysis. *Stroke*. 2020;51(3):922-930. doi:10.1161/STROKEAHA.119.027843

54. Shownkeen H, Richards CT, Buffo K, et al. Outcomes of Patients Receiving Thrombolysis in a Mobile Stroke Unit: A 4-Year Retrospective, Observational, Single-Center Study. *Prehosp Emerg Care*. Published online December 6, 2022:1-7. doi:10.1080/10903127.2022.2142991

55. Gonçalves ASO, Rohmann JL, Piccininni M, et al. Economic Evaluation of a Mobile Stroke Unit Service in Germany. *Ann Neurol*. Published online January 13, 2023. doi:10.1002/ana.26602

56. Lund UH, Stoinska-Schneider A, Larsen K, Bache KG, Robberstad B. Cost-Effectiveness of Mobile Stroke Unit Care in Norway. *Stroke*. 0(0):10.1161/STROKEAHA.121.037491. doi:10.1161/STROKEAHA.121.037491

57. Slot KB, Berge E, Dorman P, Lewis S, Dennis M, Sandercock P. Impact of functional status at six months on long term survival in patients with ischaemic stroke: prospective cohort studies. *BMJ*. 2008;336(7640):376-379. doi:10.1136/bmj.39456.688333.BE
